# Ti_4_Fe_2_C_0.82_O_0.18_

**DOI:** 10.1107/S2414314624008903

**Published:** 2024-09-30

**Authors:** Huizi Liu, Changzeng Fan, Bin Wen, Lifeng Zhang

**Affiliations:** a State Key Laboratory of Metastable Materials Science and Technology Yanshan University,Qinhuangdao 066004 People’s Republic of China; b Hebei Key Lab for Optimizing Metal Product Technology and Performance Yanshan University,Qinhuangdao 066004 People’s Republic of China; c School of Mechanical and Materials Engineering North China University of Technology,Beijing 100144 People’s Republic of China; Vienna University of Technology, Austria

**Keywords:** crystal structure, high-pressure sinter­ing, co-occupation, inter­metallics

## Abstract

The phase with composition Ti_4_Fe_2_C_0.82_O_0.18_, tetra­titanium diiron carbide oxide, was unexpectedly synthesized by high-pressure sinter­ing (HPS) of a stoichiometric mixture with nominal composition Ti_2_Fe. The Ti_4_Fe_2_C_0.82_O_0.18_ phase crystallizes in the *Fd**m* space group and can be considered as the Ti_2_Fe structure filled with C and O atoms co-occupying the same octa­hedral void [occupancy ratio 0.82 (7):0.18 (7)]. The Ti_4_Fe_2_C_0.82_O_0.18_ phase is isotypic with Ti_4_Ni_2_C and Ti_4_Fe_2_O_0.407_, and is the first example where C and O atoms co-occupy the same site in filled Ti_2_Fe structures.

## Abstract 2

Ti_4_Fe_2_C_0.82_O_0.18_ is the first example where C and O atoms co-occupy the same site in filled Ti_2_Fe structures.

## Graphical abstract

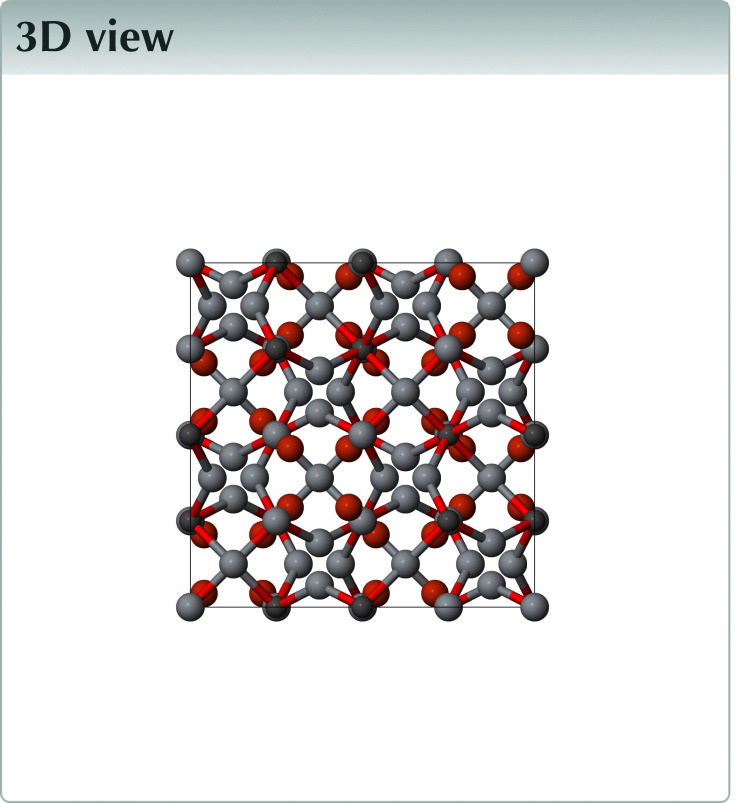

## Structure description

Inter­metallic phases usually are classified by structural or chemical similarities. For example, Duwez & Taylor (1950 ▸) investigated the crystal structure of Ti_2_Fe on the basis of X-ray powder data. They determined that the Ti_2_Fe phase crystallizes in a face centered cubic (f.c.c.) unit cell, with cell parameter *a* = 11.305 Å and with 96 atoms per unit cell. The related crystal structure of cubic Ti_4_Fe_2_O_0.407_ was refined on the basis of neutron powder diffraction, affording the cell parameter *a* = 11.3326 (5) Å in space group *Fd*
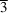
*m* with Ti on position 48 *f* and on 16 *d*, Fe on 32 *e* and O (with a site occupation factor of 0.407) on 16 *c* (Rupp & Fischer, 1988 ▸). Liu *et al.* (2024 ▸) reported the isotypic crystal structure of Ti_4_Ni_2_C [*a* = 11.3235 (8) Å] by using single-crystal X-ray diffraction (SXRD) measurements. The latter phase can be considered as a partially filled Ti_2_Ni structure with the C atom occupying an octa­hedral void. Holleck & Thummler (1967 ▸) studied a series of carbides, nitrides and oxides in ternary systems including Nb_4_Ni_2_C (*a* = 11.64 Å) and Ta_4_Ni_2_C (*a* =11.61 Å) phases. Although the title Ti_4_Fe_2_C_0.82_O_0.18_ phase is isotypic with Ti_4_Ni_2_C and Ti_4_Fe_2_O_0.407_, no detailed study has been performed so far with respect to a phase where C and O atoms co-occupy the same site. The carbon present in the crystal structure of Ti_4_Fe_2_C_0.82_O_0.18_ most likely originated from the graphite crucible used during high pressure sinter­ing (HPS), whereas oxygen may be incorporated due to surface oxidation during sample storage and subsequent HPS.

**Figure 1:**
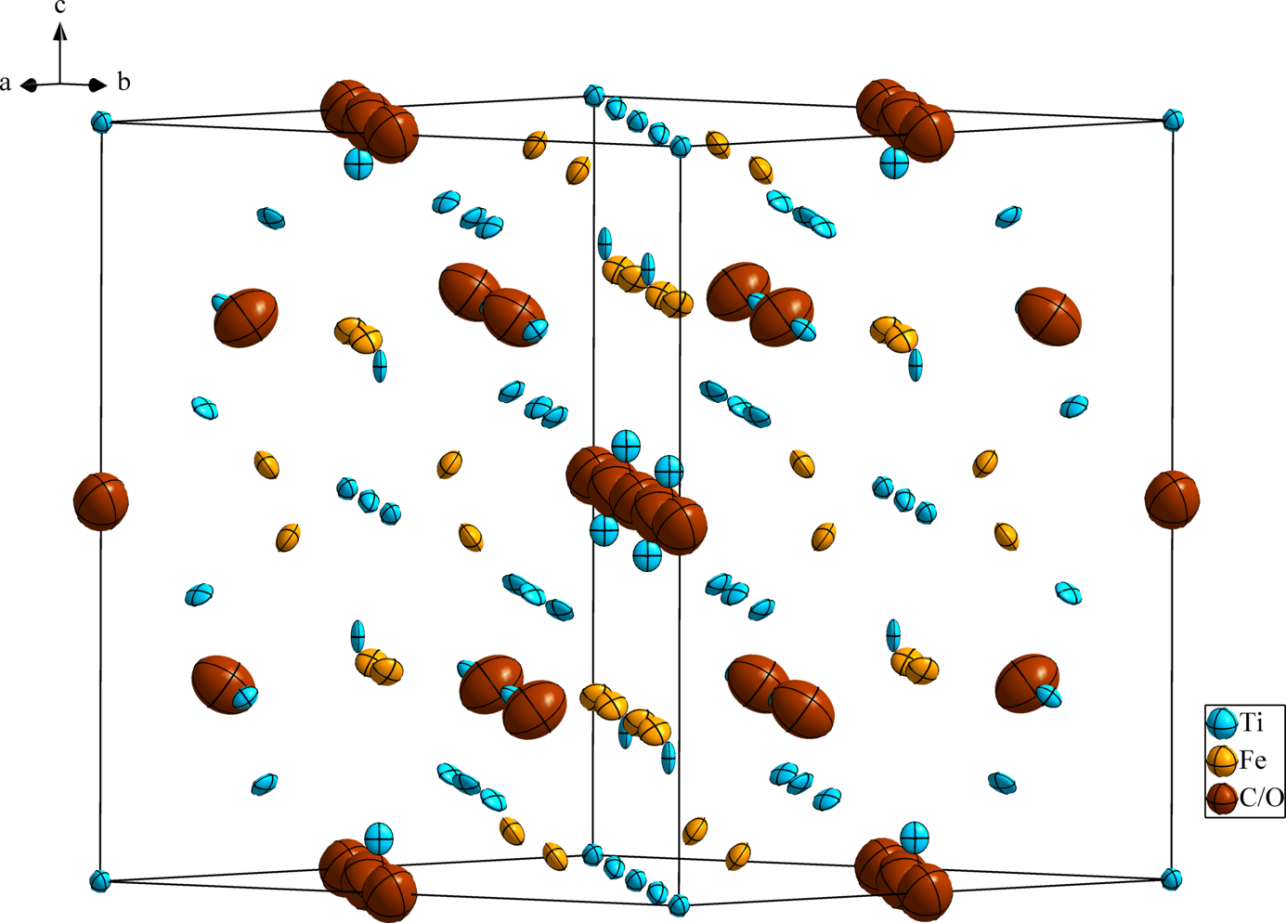
The crystal structure of Ti_4_Fe_2_C_0.82_O_0.18_, with displacement ellipsoids drawn at the 99.9% probability level.

**Figure 2:**
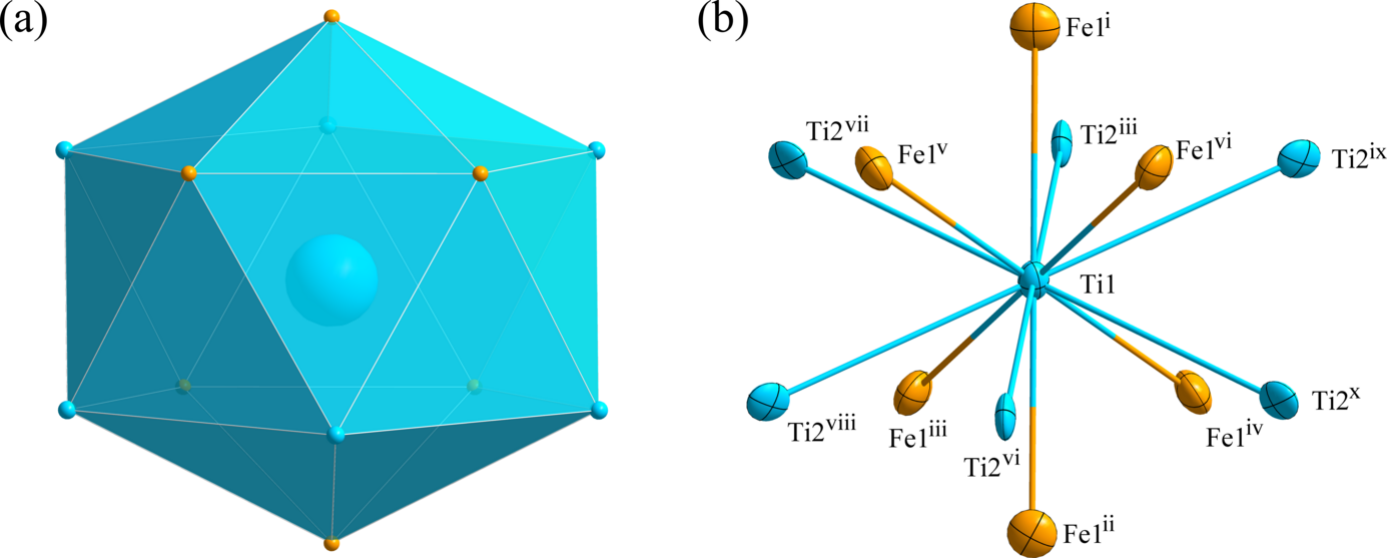
(*a*) The icosa­hedron formed around the Ti1 atom at the 16 *c* site; (*b*) the environment of the Ti1 atom with displacement ellipsoids drawn at the 99.9% probability level. [Symmetry codes: (i) −*x*, *y* − 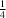, *z* − 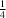; (ii) *x*, −*y* + 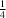, −*z* + 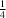; (iii) −*x* + 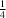, −*y* + 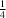, *z*; (iv) −*x* + 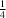, *y*, −*z* + 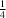; (v) *x* − 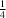, −*y*, *z* − 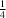; (vi) *x* − 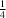, *y* − 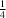, −*z*; (vii) *y* − 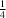, −*z*, *x* − 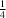; (viii) *z*, −*x* + 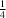, −*y* + 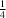; (ix) −*z*, *x* − 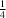, *y* − 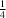; (*x*) −*y* + 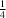, *z*, −*x* + 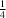.]

Ti_4_Fe_2_C_0.82_O_0.18_ crystallizes isotypically with Ti_4_Fe_2_O_0.407_ and Ti_4_Ni_2_C in a partially filled Ti_2_Fe structure in space group type *Fd*
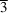
*m*. Fig. 1 ▸ shows the distribution of the atoms in the the unit cell of Ti_4_Fe_2_C_0.82_O_0.18_. The environments of the Ti1 and (C1/O1) sites are shown in Figs. 2 ▸ and 3 ▸, respectively. The Ti1 atom is situated at a position with site symmetry *.*
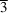
*m* (multiplicity 16, Wyckoff letter *c*). It is surrounded by six Ti2 atoms (2*.mm*, 48 *f*) and six Fe1 atoms (*.*3*m*, 32 *e*), defining the center of an icosa­hedron. The (C1/O1) atoms co-occupy a position with site symmetry .
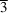
*m* (16 *d*) and center an octa­hedron defined by six Ti2 atoms. The shortest Ti1 to Ti2 separation is 2.9084 (10) Å and the shortest Ti1 to Fe1 separation is 2.4650 (4) Å; the (C1/O1)—Ti2 bond length is 2.1273 (5) Å.

## Synthesis and crystallization

Pure titanium powder (indicated purity 99.5%, 0.6312 g) and iron powder (indicated purity 99.9%, 0.3693 g) were evenly mixed according to the stoichiometric ratio of 2:1 and thoroughly ground in an agate mortar. The mixed powder was put into a 5 mm cemented carbide grinding mould and pressed into a tablet at about 6 MPa for 2 min to obtain a cylindrical block without deformations or cracks. Details of the high-pressure sinter­ing experiment using a six-anvil high-temperature and high-pressure apparatus can be found elsewhere (Liu & Fan, 2018 ▸). The sample was pressurized up to 6 GPa and heated to 1473 K for 20 minutes, cooled to 1173 K, held at the temperature for 1 h, and then the furnace power was turned off to rapidly cool to room temperature. Different phases were isolated from two samples from the same batch. Ti_4_Fe_2_C_0.82_O_0.18_ originated from sample 1, together with TiFe. The refined chemical formula of Ti_4_Fe_2_C_0.82_O_0.18_ from sample 1 is in accordance with the complementary EDX results (see Table S1 of the electronic supporting information, ESI). Another phase with very similar refined composition, Ti_4_Fe_2_C_0.87_O_0.13_, was isolated from sample 2, and its composition is also in accordance with the complementary EDX results (see Table S2 of the ESI). Different options for refinements for the two phases Ti_4_Fe_2_C_1-δ_O_δ_ (δ = 0.18; δ = 0.13) are listed in Table S3 of the ESI. The crystal structures of Ti_4_Fe_2_C_0.82_O_0.18_ and Ti_4_Fe_2_C_0.87_O_0.13_ are very similar, just different in atomic proportions at the 16 *d* site, so the Ti_4_Fe_2_C_0.82_O_0.18_ phase was selected for the current report. Structural data for Ti_4_Fe_2_C_0.87_O_0.13_ can be found in Table S4 of the ESI.

## Refinement

Crystal data, data collection and structure refinement details of Ti_4_Fe_2_C_0.82_O_0.18_ are summarized in Table 1 ▸. The labeling scheme and atomic coordinates of Ti_4_Fe_2_C_0.82_O_0.18_ were adapted from Ti_4_Ni_2_C for better comparison (Liu *et al.*, 2024 ▸). The 16 *d* site is co-occupied by C and O atoms, with site occupancies refined to 0.82 (7) for C1 and 0.18 (7) for O1, assuming full occupancy. Both atoms were refined with the same displacement parameters. The maximum and minimum residual electron densities in the final difference map are located 1.16 Å from site Fe1 and 1.67 Å from Fe1, respectively.

## Supplementary Material

Crystal structure: contains datablock(s) I. DOI: 10.1107/S2414314624008903/wm5714sup1.cif

Structure factors: contains datablock(s) I. DOI: 10.1107/S2414314624008903/wm5714Isup6.hkl

Supporting information file. DOI: 10.1107/S2414314624008903/wm5714sup7.docx

CCDC reference: 2383278

Additional supporting information:  crystallographic information; 3D view; checkCIF report

## Figures and Tables

**Figure 3:**
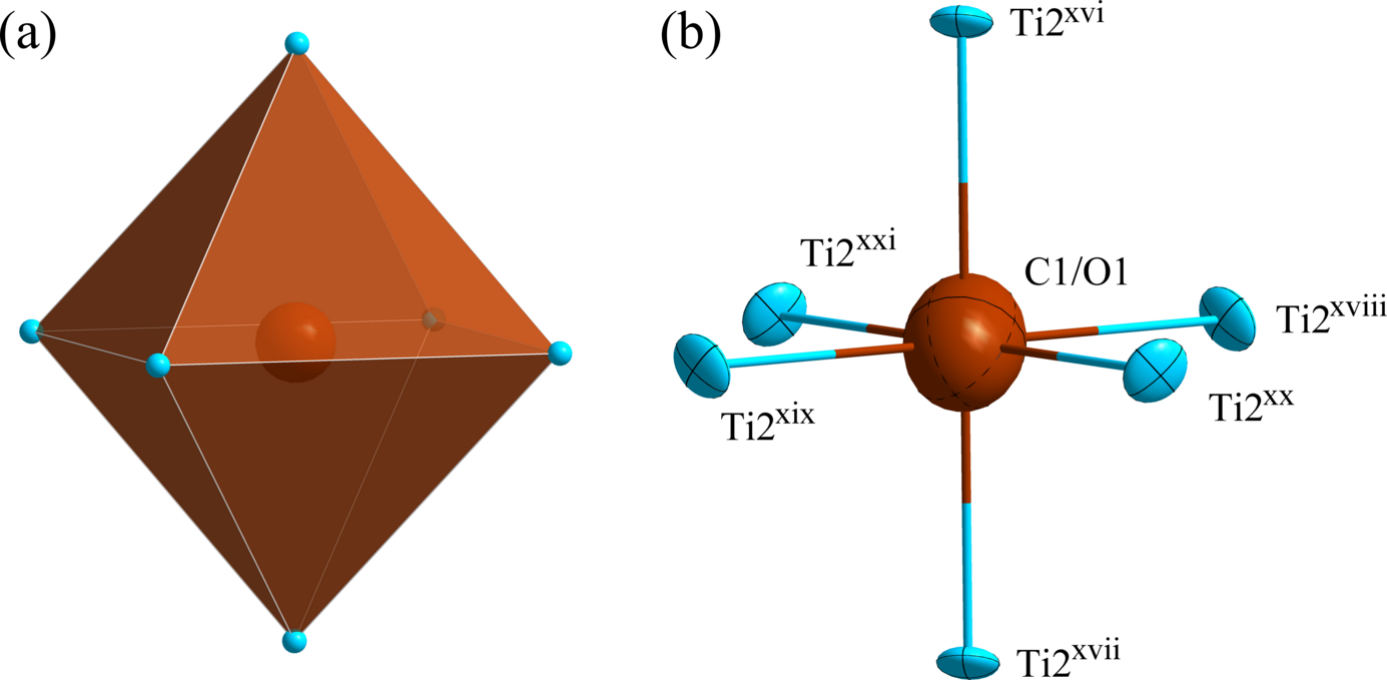
(*a*) The octa­hedron formed around the (C1/O1) atoms at the 16 *d* site; (*b*) the environment of the (C1/O1) atoms with displacement ellipsoids drawn at the 99.9% probability level. [Symmetry codes: (xvi) −*z* + 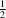, −*x* + 1, −*y* + 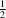; (xvii) *z* + 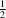, *x*, *y* + 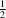; (xviii) *x*, *y* + 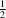, *z* + 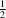; (xix) −*x* + 1, −*y* + 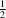, −*z* + 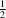; (xx) −*y* + 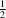, −*z* + 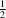, −*x* + 1; (xxi) *y* + 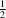, *z* + 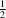, *x*.]

**Table 1:** Experimental details

Crystal data	
Chemical formula	Ti_4_Fe_2_C_0.82_O_0.18_
*M*_r_	316.02
Crystal system, space group	Cubic, *Fd* 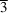 *m*
Temperature (K)	296
*a* (Å)	11.323 (4)
*V* (Å^3^)	1451.6 (3)
*Z*	16
Radiation type	Mo *K*α
μ (mm^−1^)	15.91
Crystal size (mm)	0.10 × 0.06 × 0.06

Data collection	
Diffractometer	Bruker D8 Venture Photon 100 CMOS
Absorption correction	Multi-scan (*SADABS*; Krause *et al.*, 2015 ▸)
*T*_min_, *T*_max_	0.429, 0.746
No. of measured, independent and observed [*I* > 2σ(*I*)] reflections	5363, 102, 82
*R*_int_	0.155
(sin θ/λ)_max_ (Å^−1^)	0.643

Refinement	
*R*[*F*^2^ > 2σ(*F*^2^)], *wR*(*F*^2^), *S*	0.022, 0.040, 1.14
No. of reflections	102
No. of parameters	13
Δρ_max_, Δρ_min_ (e Å^−3^)	0.58, −0.83

Computer programs: *APEX3* and *SAINT* (Bruker, 2015 ▸), *SHELXT* (Sheldrick, 2015*a* ▸), *SHELXL* (Sheldrick, 2015*b* ▸), *DIAMOND* (Brandenburg & Putz, 2017 ▸) and *publCIF* (Westrip, 2010 ▸).

## full crystallographic data

### Tetratitanium diiron carbide oxide Crystal data

**Table:**

Ti_4_Fe_2_C_0.82_O_0.18_	Mo *K*α radiation, λ = 0.71073 Å
*M**_r_* = 316.02	Cell parameters from 1378 reflections
Cubic, *F**d*3*m*	θ = 3.1–26.7°
*a* = 11.323 (4) Å	µ = 15.91 mm^−^^1^
*V* = 1451.6 (3) Å^3^	*T* = 296 K
*Z* = 16	Lump, gray
*F*(000) = 2342	0.10 × 0.06 × 0.06 mm
*D*_x_ = 5.784 Mg m^−^^3^	

### Tetratitanium diiron carbide oxide Data collection

**Table:**

Bruker D8 Venture Photon 100 CMOS diffractometer	82 reflections with *I* > 2σ(*I*)
phi and ω scans	*R*_int_ = 0.155
Absorption correction: multi-scan (SADABS; Krause *et al.*, 2015)	θ_max_ = 27.2°, θ_min_ = 3.1°
*T*_min_ = 0.429, *T*_max_ = 0.746	*h* = −14→14
5363 measured reflections	*k* = −13→14
102 independent reflections	*l* = −14→14

### Tetratitanium diiron carbide oxide Refinement

**Table:**

Refinement on *F*^2^	13 parameters
Least-squares matrix: full	0 restraints
*R*[*F*^2^ > 2σ(*F*^2^)] = 0.022	*w* = 1/[σ^2^(*F*_o_^2^) + (0.0069*P*)^2^ + 29.2966*P*] where *P* = (*F*_o_^2^ + 2*F*_c_^2^)/3
*wR*(*F*^2^) = 0.040	(Δ/σ)_max_ < 0.001
*S* = 1.14	Δρ_max_ = 0.58 e Å^−^^3^
102 reflections	Δρ_min_ = −0.83 e Å^−^^3^

### Tetratitanium diiron carbide oxide Special details

**Table:**

Geometry. All esds (except the esd in the dihedral angle between two l.s. planes) are estimated using the full covariance matrix. The cell esds are taken into account individually in the estimation of esds in distances, angles and torsion angles; correlations between esds in cell parameters are only used when they are defined by crystal symmetry. An approximate (isotropic) treatment of cell esds is used for estimating esds involving l.s. planes.

### Tetratitanium diiron carbide oxide Fractional atomic coordinates and isotropic or equivalent isotropic displacement parameters (Å^2^)

**Table:**

	*x*	*y*	*z*	*U*_iso_*/*U*_eq_	Occ. (<1)
Ti1	0.000000	0.000000	0.000000	0.0018 (5)	
Fe1	0.21037 (6)	0.21037 (6)	0.21037 (6)	0.0028 (4)	
Ti2	0.43636 (12)	0.125000	0.125000	0.0021 (4)	
C1	0.500000	0.500000	0.500000	0.012 (4)	0.82 (7)
O1	0.500000	0.500000	0.500000	0.012 (4)	0.18 (7)

### Tetratitanium diiron carbide oxide Atomic displacement parameters (Å^2^)

**Table:**

	*U*^11^	*U*^22^	*U*^33^	*U*^12^	*U*^13^	*U*^23^
Ti1	0.0018 (5)	0.0018 (5)	0.0018 (5)	0.0004 (6)	0.0004 (6)	0.0004 (6)
Fe1	0.0028 (4)	0.0028 (4)	0.0028 (4)	−0.0007 (3)	−0.0007 (3)	−0.0007 (3)
Ti2	0.0033 (8)	0.0016 (5)	0.0016 (5)	0.000	0.000	−0.0010 (5)
C1	0.012 (4)	0.012 (4)	0.012 (4)	0.002 (4)	0.002 (4)	0.002 (4)
O1	0.012 (4)	0.012 (4)	0.012 (4)	0.002 (4)	0.002 (4)	0.002 (4)

### Tetratitanium diiron carbide oxide Geometric parameters (Å, º)

**Table:**

Ti1—Fe1^i^	2.4650 (4)	Fe1—Ti2^xiii^	2.6501 (10)
Ti1—Fe1^ii^	2.4650 (4)	Fe1—Fe1^iii^	2.734 (2)
Ti1—Fe1^iii^	2.4650 (4)	Fe1—Fe1^ii^	2.734 (2)
Ti1—Fe1^iv^	2.4650 (4)	Fe1—Fe1^iv^	2.734 (2)
Ti1—Fe1^v^	2.4650 (4)	Fe1—Ti2	2.9011 (12)
Ti1—Fe1^vi^	2.4650 (4)	Fe1—Ti2^xiv^	2.9011 (12)
Ti1—Ti2^vii^	2.9084 (10)	Fe1—Ti2^xv^	2.9011 (12)
Ti1—Ti2^iii^	2.9084 (10)	Ti2—C1^xvi^	2.1273 (5)
Ti1—Ti2^vi^	2.9084 (10)	Ti2—C1^xvii^	2.1273 (5)
Ti1—Ti2^viii^	2.9084 (10)	Ti2—Ti2^xviii^	2.9963 (6)
Ti1—Ti2^ix^	2.9084 (10)	Ti2—Ti2^xix^	2.9963 (6)
Ti1—Ti2^x^	2.9084 (10)	Ti2—Ti2^xi^	2.9963 (6)
Fe1—Ti2^xi^	2.6501 (10)	Ti2—Ti2^xii^	2.9963 (6)
Fe1—Ti2^xii^	2.6501 (10)		
			
Fe1^i^—Ti1—Fe1^ii^	180.0	Ti2^xi^—Fe1—Ti2^xiv^	65.152 (14)
Fe1^i^—Ti1—Fe1^iii^	112.64 (4)	Ti2^xii^—Fe1—Ti2^xiv^	124.00 (6)
Fe1^ii^—Ti1—Fe1^iii^	67.36 (4)	Ti2^xiii^—Fe1—Ti2^xiv^	65.152 (14)
Fe1^i^—Ti1—Fe1^iv^	112.64 (4)	Fe1^iii^—Fe1—Ti2^xiv^	112.83 (2)
Fe1^ii^—Ti1—Fe1^iv^	67.36 (4)	Fe1^ii^—Fe1—Ti2^xiv^	112.83 (2)
Fe1^iii^—Ti1—Fe1^iv^	67.36 (4)	Fe1^iv^—Fe1—Ti2^xiv^	61.89 (3)
Fe1^i^—Ti1—Fe1^v^	67.36 (4)	Ti2—Fe1—Ti2^xiv^	118.474 (12)
Fe1^ii^—Ti1—Fe1^v^	112.64 (4)	Ti1^ii^—Fe1—Ti2^xv^	65.047 (6)
Fe1^iii^—Ti1—Fe1^v^	112.64 (4)	Ti1^iii^—Fe1—Ti2^xv^	166.80 (5)
Fe1^iv^—Ti1—Fe1^v^	180.0	Ti1^iv^—Fe1—Ti2^xv^	65.047 (6)
Fe1^i^—Ti1—Fe1^vi^	67.36 (4)	Ti2^xi^—Fe1—Ti2^xv^	124.00 (6)
Fe1^ii^—Ti1—Fe1^vi^	112.64 (4)	Ti2^xii^—Fe1—Ti2^xv^	65.152 (14)
Fe1^iii^—Ti1—Fe1^vi^	180.0	Ti2^xiii^—Fe1—Ti2^xv^	65.152 (14)
Fe1^iv^—Ti1—Fe1^vi^	112.64 (4)	Fe1^iii^—Fe1—Ti2^xv^	61.89 (3)
Fe1^v^—Ti1—Fe1^vi^	67.36 (4)	Fe1^ii^—Fe1—Ti2^xv^	112.83 (2)
Fe1^i^—Ti1—Ti2^vii^	64.739 (17)	Fe1^iv^—Fe1—Ti2^xv^	112.83 (2)
Fe1^ii^—Ti1—Ti2^vii^	115.261 (17)	Ti2—Fe1—Ti2^xv^	118.474 (12)
Fe1^iii^—Ti1—Ti2^vii^	58.40 (3)	Ti2^xiv^—Fe1—Ti2^xv^	118.474 (12)
Fe1^iv^—Ti1—Ti2^vii^	115.261 (17)	C1^xvi^—Ti2—C1^xvii^	140.40 (7)
Fe1^v^—Ti1—Ti2^vii^	64.739 (17)	C1^xvi^—Ti2—Fe1^xx^	88.009 (14)
Fe1^vi^—Ti1—Ti2^vii^	121.60 (3)	C1^xvii^—Ti2—Fe1^xx^	88.009 (14)
Fe1^i^—Ti1—Ti2^iii^	58.40 (3)	C1^xvi^—Ti2—Fe1^xxi^	88.009 (14)
Fe1^ii^—Ti1—Ti2^iii^	121.60 (3)	C1^xvii^—Ti2—Fe1^xxi^	88.009 (14)
Fe1^iii^—Ti1—Ti2^iii^	64.739 (17)	Fe1^xx^—Ti2—Fe1^xxi^	168.22 (7)
Fe1^iv^—Ti1—Ti2^iii^	64.739 (17)	C1^xvi^—Ti2—Fe1^ii^	137.91 (5)
Fe1^v^—Ti1—Ti2^iii^	115.261 (17)	C1^xvii^—Ti2—Fe1^ii^	81.68 (3)
Fe1^vi^—Ti1—Ti2^iii^	115.261 (17)	Fe1^xx^—Ti2—Fe1^ii^	95.19 (3)
Ti2^vii^—Ti1—Ti2^iii^	62.010 (9)	Fe1^xxi^—Ti2—Fe1^ii^	95.19 (3)
Fe1^i^—Ti1—Ti2^vi^	121.60 (3)	C1^xvi^—Ti2—Fe1	81.69 (3)
Fe1^ii^—Ti1—Ti2^vi^	58.40 (3)	C1^xvii^—Ti2—Fe1	137.91 (5)
Fe1^iii^—Ti1—Ti2^vi^	115.261 (17)	Fe1^xx^—Ti2—Fe1	95.19 (3)
Fe1^iv^—Ti1—Ti2^vi^	115.261 (17)	Fe1^xxi^—Ti2—Fe1	95.19 (3)
Fe1^v^—Ti1—Ti2^vi^	64.739 (17)	Fe1^ii^—Ti2—Fe1	56.23 (6)
Fe1^vi^—Ti1—Ti2^vi^	64.739 (17)	C1^xvi^—Ti2—Ti1^iv^	104.226 (19)
Ti2^vii^—Ti1—Ti2^vi^	117.990 (9)	C1^xvii^—Ti2—Ti1^iv^	104.226 (19)
Ti2^iii^—Ti1—Ti2^vi^	180.0	Fe1^xx^—Ti2—Ti1^iv^	52.40 (2)
Fe1^i^—Ti1—Ti2^viii^	115.261 (17)	Fe1^xxi^—Ti2—Ti1^iv^	139.38 (5)
Fe1^ii^—Ti1—Ti2^viii^	64.739 (17)	Fe1^ii^—Ti2—Ti1^iv^	50.21 (2)
Fe1^iii^—Ti1—Ti2^viii^	64.739 (17)	Fe1—Ti2—Ti1^iv^	50.21 (2)
Fe1^iv^—Ti1—Ti2^viii^	121.60 (3)	C1^xvi^—Ti2—Ti1^iii^	104.226 (19)
Fe1^v^—Ti1—Ti2^viii^	58.40 (3)	C1^xvii^—Ti2—Ti1^iii^	104.226 (19)
Fe1^vi^—Ti1—Ti2^viii^	115.261 (17)	Fe1^xx^—Ti2—Ti1^iii^	139.38 (5)
Ti2^vii^—Ti1—Ti2^viii^	62.010 (9)	Fe1^xxi^—Ti2—Ti1^iii^	52.40 (2)
Ti2^iii^—Ti1—Ti2^viii^	117.990 (9)	Fe1^ii^—Ti2—Ti1^iii^	50.21 (2)
Ti2^vi^—Ti1—Ti2^viii^	62.010 (9)	Fe1—Ti2—Ti1^iii^	50.21 (2)
Fe1^i^—Ti1—Ti2^ix^	64.739 (17)	Ti1^iv^—Ti2—Ti1^iii^	86.98 (4)
Fe1^ii^—Ti1—Ti2^ix^	115.261 (17)	C1^xvi^—Ti2—Ti2^xviii^	149.47 (3)
Fe1^iii^—Ti1—Ti2^ix^	115.261 (17)	C1^xvii^—Ti2—Ti2^xviii^	45.23 (2)
Fe1^iv^—Ti1—Ti2^ix^	58.40 (3)	Fe1^xx^—Ti2—Ti2^xviii^	121.68 (3)
Fe1^v^—Ti1—Ti2^ix^	121.60 (3)	Fe1^xxi^—Ti2—Ti2^xviii^	61.47 (2)
Fe1^vi^—Ti1—Ti2^ix^	64.739 (17)	Fe1^ii^—Ti2—Ti2^xviii^	53.38 (3)
Ti2^vii^—Ti1—Ti2^ix^	117.990 (9)	Fe1—Ti2—Ti2^xviii^	100.81 (3)
Ti2^iii^—Ti1—Ti2^ix^	62.010 (9)	Ti1^iv^—Ti2—Ti2^xviii^	100.29 (4)
Ti2^vi^—Ti1—Ti2^ix^	117.990 (9)	Ti1^iii^—Ti2—Ti2^xviii^	58.995 (4)
Ti2^viii^—Ti1—Ti2^ix^	180.0	C1^xvi^—Ti2—Ti2^xix^	149.47 (3)
Fe1^i^—Ti1—Ti2^x^	115.261 (17)	C1^xvii^—Ti2—Ti2^xix^	45.23 (2)
Fe1^ii^—Ti1—Ti2^x^	64.739 (17)	Fe1^xx^—Ti2—Ti2^xix^	61.47 (2)
Fe1^iii^—Ti1—Ti2^x^	121.60 (3)	Fe1^xxi^—Ti2—Ti2^xix^	121.68 (3)
Fe1^iv^—Ti1—Ti2^x^	64.739 (17)	Fe1^ii^—Ti2—Ti2^xix^	53.38 (3)
Fe1^v^—Ti1—Ti2^x^	115.261 (17)	Fe1—Ti2—Ti2^xix^	100.81 (3)
Fe1^vi^—Ti1—Ti2^x^	58.40 (3)	Ti1^iv^—Ti2—Ti2^xix^	58.995 (4)
Ti2^vii^—Ti1—Ti2^x^	180.0	Ti1^iii^—Ti2—Ti2^xix^	100.29 (4)
Ti2^iii^—Ti1—Ti2^x^	117.990 (9)	Ti2^xviii^—Ti2—Ti2^xix^	60.54 (6)
Ti2^vi^—Ti1—Ti2^x^	62.010 (9)	C1^xvi^—Ti2—Ti2^xi^	45.23 (2)
Ti2^viii^—Ti1—Ti2^x^	117.990 (9)	C1^xvii^—Ti2—Ti2^xi^	149.47 (3)
Ti2^ix^—Ti1—Ti2^x^	62.010 (9)	Fe1^xx^—Ti2—Ti2^xi^	121.68 (3)
Ti1^ii^—Fe1—Ti1^iii^	108.58 (3)	Fe1^xxi^—Ti2—Ti2^xi^	61.47 (2)
Ti1^ii^—Fe1—Ti1^iv^	108.58 (3)	Fe1^ii^—Ti2—Ti2^xi^	100.81 (3)
Ti1^iii^—Fe1—Ti1^iv^	108.58 (3)	Fe1—Ti2—Ti2^xi^	53.38 (3)
Ti1^ii^—Fe1—Ti2^xi^	124.77 (2)	Ti1^iv^—Ti2—Ti2^xi^	100.29 (4)
Ti1^iii^—Fe1—Ti2^xi^	69.20 (3)	Ti1^iii^—Ti2—Ti2^xi^	58.995 (4)
Ti1^iv^—Fe1—Ti2^xi^	124.77 (2)	Ti2^xviii^—Ti2—Ti2^xi^	112.60 (3)
Ti1^ii^—Fe1—Ti2^xii^	124.77 (2)	Ti2^xix^—Ti2—Ti2^xi^	153.19 (5)
Ti1^iii^—Fe1—Ti2^xii^	124.77 (2)	C1^xvi^—Ti2—Ti2^xii^	45.23 (2)
Ti1^iv^—Fe1—Ti2^xii^	69.20 (3)	C1^xvii^—Ti2—Ti2^xii^	149.47 (3)
Ti2^xi^—Fe1—Ti2^xii^	69.49 (5)	Fe1^xx^—Ti2—Ti2^xii^	61.47 (2)
Ti1^ii^—Fe1—Ti2^xiii^	69.20 (3)	Fe1^xxi^—Ti2—Ti2^xii^	121.68 (3)
Ti1^iii^—Fe1—Ti2^xiii^	124.77 (2)	Fe1^ii^—Ti2—Ti2^xii^	100.81 (3)
Ti1^iv^—Fe1—Ti2^xiii^	124.77 (2)	Fe1—Ti2—Ti2^xii^	53.38 (3)
Ti2^xi^—Fe1—Ti2^xiii^	69.49 (5)	Ti1^iv^—Ti2—Ti2^xii^	58.995 (4)
Ti2^xii^—Fe1—Ti2^xiii^	69.49 (5)	Ti1^iii^—Ti2—Ti2^xii^	100.29 (4)
Ti1^ii^—Fe1—Fe1^iii^	56.32 (2)	Ti2^xviii^—Ti2—Ti2^xii^	153.19 (5)
Ti1^iii^—Fe1—Fe1^iii^	104.92 (3)	Ti2^xix^—Ti2—Ti2^xii^	112.60 (3)
Ti1^iv^—Fe1—Fe1^iii^	56.32 (2)	Ti2^xi^—Ti2—Ti2^xii^	60.54 (6)
Ti2^xi^—Fe1—Fe1^iii^	174.11 (3)	Ti2^xxii^—C1—Ti2^xxiii^	180.0
Ti2^xii^—Fe1—Fe1^iii^	115.14 (3)	Ti2^xxii^—C1—Ti2^xxiv^	89.54 (5)
Ti2^xiii^—Fe1—Fe1^iii^	115.14 (3)	Ti2^xxiii^—C1—Ti2^xxiv^	90.46 (5)
Ti1^ii^—Fe1—Fe1^ii^	104.92 (3)	Ti2^xxii^—C1—Ti2^xxv^	90.46 (5)
Ti1^iii^—Fe1—Fe1^ii^	56.32 (2)	Ti2^xxiii^—C1—Ti2^xxv^	89.54 (5)
Ti1^iv^—Fe1—Fe1^ii^	56.32 (2)	Ti2^xxiv^—C1—Ti2^xxv^	180.0
Ti2^xi^—Fe1—Fe1^ii^	115.14 (3)	Ti2^xxii^—C1—Ti2^xxvi^	90.46 (5)
Ti2^xii^—Fe1—Fe1^ii^	115.14 (3)	Ti2^xxiii^—C1—Ti2^xxvi^	89.54 (5)
Ti2^xiii^—Fe1—Fe1^ii^	174.11 (3)	Ti2^xxiv^—C1—Ti2^xxvi^	89.54 (5)
Fe1^iii^—Fe1—Fe1^ii^	60.0	Ti2^xxv^—C1—Ti2^xxvi^	90.46 (5)
Ti1^ii^—Fe1—Fe1^iv^	56.32 (2)	Ti2^xxii^—C1—Ti2^xxvii^	89.54 (5)
Ti1^iii^—Fe1—Fe1^iv^	56.32 (2)	Ti2^xxiii^—C1—Ti2^xxvii^	90.46 (5)
Ti1^iv^—Fe1—Fe1^iv^	104.92 (3)	Ti2^xxiv^—C1—Ti2^xxvii^	90.46 (5)
Ti2^xi^—Fe1—Fe1^iv^	115.14 (3)	Ti2^xxv^—C1—Ti2^xxvii^	89.54 (5)
Ti2^xii^—Fe1—Fe1^iv^	174.11 (3)	Ti2^xxvi^—C1—Ti2^xxvii^	180.0
Ti2^xiii^—Fe1—Fe1^iv^	115.14 (3)	Ti2^xxii^—O1—Ti2^xxiii^	180.0
Fe1^iii^—Fe1—Fe1^iv^	60.0	Ti2^xxii^—O1—Ti2^xxiv^	89.54 (5)
Fe1^ii^—Fe1—Fe1^iv^	60.0	Ti2^xxiii^—O1—Ti2^xxiv^	90.46 (5)
Ti1^ii^—Fe1—Ti2	166.80 (5)	Ti2^xxii^—O1—Ti2^xxv^	90.46 (5)
Ti1^iii^—Fe1—Ti2	65.047 (6)	Ti2^xxiii^—O1—Ti2^xxv^	89.54 (5)
Ti1^iv^—Fe1—Ti2	65.047 (6)	Ti2^xxiv^—O1—Ti2^xxv^	180.0
Ti2^xi^—Fe1—Ti2	65.152 (14)	Ti2^xxii^—O1—Ti2^xxvi^	90.46 (5)
Ti2^xii^—Fe1—Ti2	65.152 (14)	Ti2^xxiii^—O1—Ti2^xxvi^	89.54 (5)
Ti2^xiii^—Fe1—Ti2	124.00 (6)	Ti2^xxiv^—O1—Ti2^xxvi^	89.54 (5)
Fe1^iii^—Fe1—Ti2	112.83 (2)	Ti2^xxv^—O1—Ti2^xxvi^	90.46 (5)
Fe1^ii^—Fe1—Ti2	61.89 (3)	Ti2^xxii^—O1—Ti2^xxvii^	89.54 (5)
Fe1^iv^—Fe1—Ti2	112.83 (2)	Ti2^xxiii^—O1—Ti2^xxvii^	90.46 (5)
Ti1^ii^—Fe1—Ti2^xiv^	65.047 (6)	Ti2^xxiv^—O1—Ti2^xxvii^	90.46 (5)
Ti1^iii^—Fe1—Ti2^xiv^	65.047 (6)	Ti2^xxv^—O1—Ti2^xxvii^	89.54 (5)
Ti1^iv^—Fe1—Ti2^xiv^	166.80 (5)	Ti2^xxvi^—O1—Ti2^xxvii^	180.0

Symmetry codes: (i) −*x*, *y*−1/4, *z*−1/4; (ii) *x*, −*y*+1/4, −*z*+1/4; (iii) −*x*+1/4, −*y*+1/4, *z*; (iv) −*x*+1/4, *y*, −*z*+1/4; (v) *x*−1/4, −*y*, *z*−1/4; (vi) *x*−1/4, *y*−1/4, −*z*; (vii) *y*−1/4, −*z*, *x*−1/4; (viii) *z*, −*x*+1/4, −*y*+1/4; (ix) −*z*, *x*−1/4, *y*−1/4; (x) −*y*+1/4, *z*, −*x*+1/4; (xi) *y*+1/4, −*z*+1/2, *x*−1/4; (xii) −*z*+1/2, *x*−1/4, *y*+1/4; (xiii) *x*−1/4, *y*+1/4, −*z*+1/2; (xiv) *z*, *x*, *y*; (xv) *y*, *z*, *x*; (xvi) *x*, −*y*+3/4, −*z*+3/4; (xvii) *x*, *y*−1/2, *z*−1/2; (xviii) −*z*+1/2, −*x*+1/2, −*y*; (xix) −*y*+1/2, −*z*, −*x*+1/2; (xx) *x*+1/4, *y*−1/4, −*z*+1/2; (xxi) *x*+1/4, −*y*+1/2, *z*−1/4; (xxii) −*z*+1/2, −*x*+1, −*y*+1/2; (xxiii) *z*+1/2, *x*, *y*+1/2; (xxiv) *x*, *y*+1/2, *z*+1/2; (xxv) −*x*+1, −*y*+1/2, −*z*+1/2; (xxvi) −*y*+1/2, −*z*+1/2, −*x*+1; (xxvii) *y*+1/2, *z*+1/2, *x*.
