# Supplementary material for: Ti4Fe2C0.82O0.18
Source: IUCrdata. 2024 Sep 30;9(Pt 9):x240890. doi: 10.1107/S2414314624008903 (PMC11451028; doi:10.1107/S2414314624008903)
Supplement: Supplementary file 3 [file x-09-x240890-sup7.docx]

**SUPPLEMENTARY MATERIALS:**

**Crystal structure of Ti_4_Fe_2_C_0.82_O_0.18_**

**Huizi Liu**^a^**,** **Changzeng Fan**^a,b,^***, Bin Wen**^a^**,** and **Lifeng Zhang** ^a,c^

^a^ State Key Laboratory of Metastable Materials Science and Technology, Yanshan University,

Qinhuangdao 066004, People’s Republic of China

^b^ Hebei Key Lab for Optimizing Metal Product Technology and Performance, Yanshan University, Qinhuangdao, Hebei 066004, People’s Republic of China

^c^ School of Mechanical and Materials Engineering, North China University of Technology, Beijing 100144, People's Republic of China

*Correspondence email: [chzfan@ysu.edu.cn](mailto:chzfan@ysu.edu.cn)

The chemical compositions were examined quantitatively by energy dispersive X-ray spectroscopy (EDX) analysis attached to a Hitachi S-3400N SEM for the purpose of guiding the crystal structure refinement. The examined points and areas of sample 1 (sample 2) are designated in Fig. S1 (Fig. S2), and the corresponding results are listed in Table S1 (Table S2). The EDX results of sample 1 showed that the ratio of Ti : Fe is less than 4 : 2 due to the existence of TiFe phase. The EDX results of two samples showed that samples contained both carbon and oxygen elements. In addition, the conductive adhesives and glues may also result in the detected impurity elements of carbon and oxygen. The deviation relative to the results of refinement of chemical composition is probably caused by the tilt of the single crystal surface to the incident beam. For ease of reading, the atomic ratio of Ti, Fe, C and O was calculated and shown in the last column of Table S1 (Table S2).


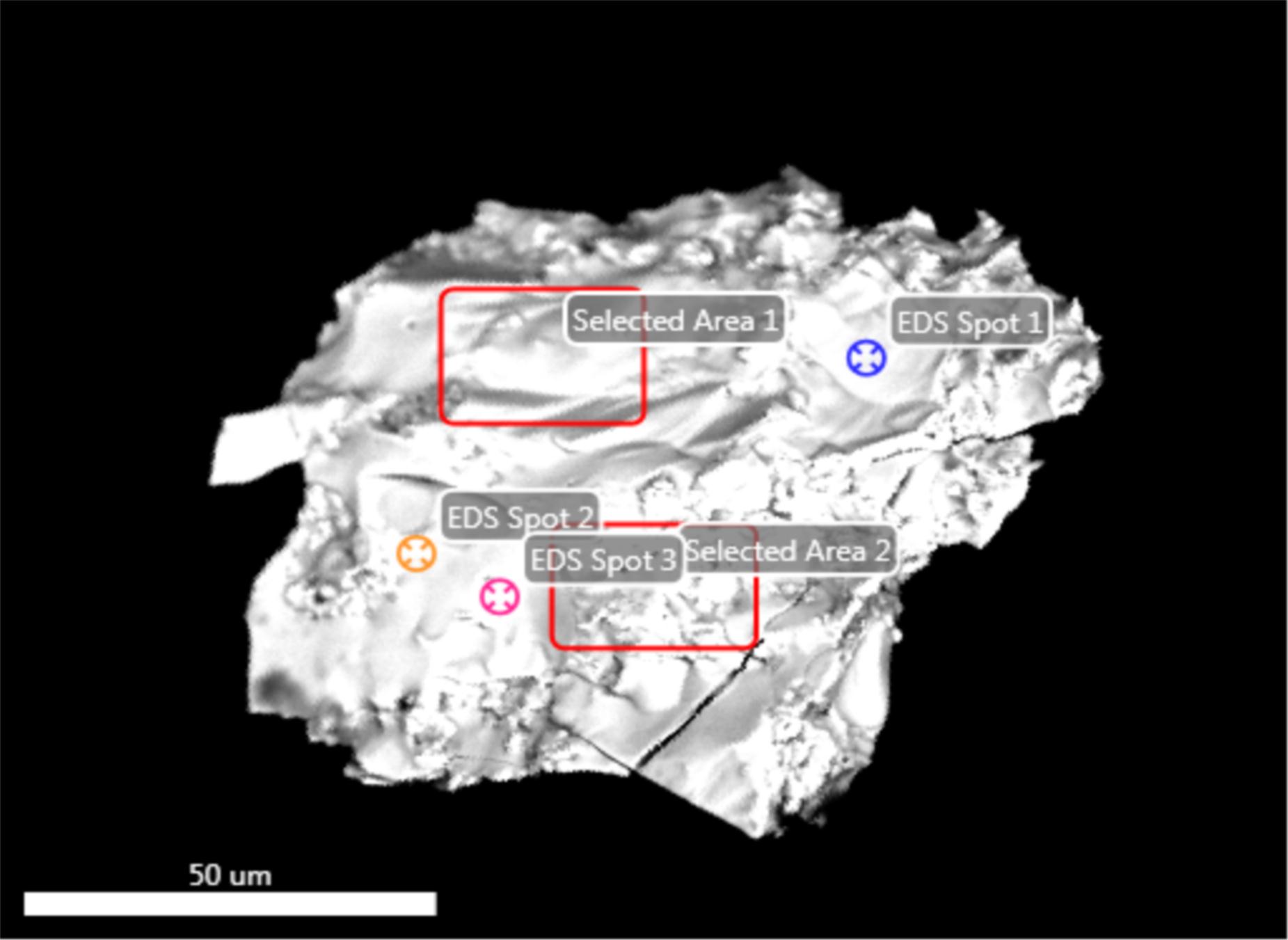


Fig. S1 Sample 1 with selected spots and areas for EDX analysis

**Table S1 EDX results for selected points and areas as designated in Fig. S1**

|  | Element | Weight (%) | Atomic (%) | Error (%) | Ti : Fe : C : O |
| --- | --- | --- | --- | --- | --- |
| Spot1 | TiK | 61.77 | 65.32 | 1.69 | 3.77:2:×:× |
|  | FeK | 38.23 | 34.68 | 2.65 |  |
| Spot2 | TiK | 56.02 | 44.12 | 1.62 | 3.92:2:2.96:× |
|  | FeK  C K | 33.36  10.62 | 22.54  33.35 | 2.55  8.87 |  |
| Spot3 | TiK | 57.25 | 47.87 | 1.64 | 3.86:2:2.21:× |
|  | FeK  C K | 34.55  8.20 | 24.78  27.35 | 2.54  9.12 |  |
| Area1 | TiK | 51.24 | 36.61 | 1.61 | 3.84:2:2.77:1.88 |
|  | FeK | 31.11 | 19.06 | 2.49 |  |
|  | C K  O K | 9.27  8.38 | 26.42  17.91 | 8.86  11.36 |  |
| Area2 | TiK | 51.62 | 36.47 | 1.60 | 3.94:2:3.16:1.70 |
|  | FeK | 30.54 | 18.51 | 2.49 |  |
|  | C K  O K | 10.39  7.44 | 29.28  15.74 | 8.76  11.47 |  |

Ti_4_Fe_2_C_0.87_O_0.13_ phase come from sample 2, where diffraction points can be clearly divided into three data sets: two data sets of Ti_4_Fe_2_C_0.87_O_0.13_ phase and one data set of TiFe phase. Among the two data sets of Ti_4_Fe_2_C_0.87_O_0.13_ phase lattice, the exact crystal structure of Ti_4_Fe_2_C_0.87_O_0.13_ phase comes from one, and the other data set is unsatisfactory due to the presence of NPD atoms during the refinement process.


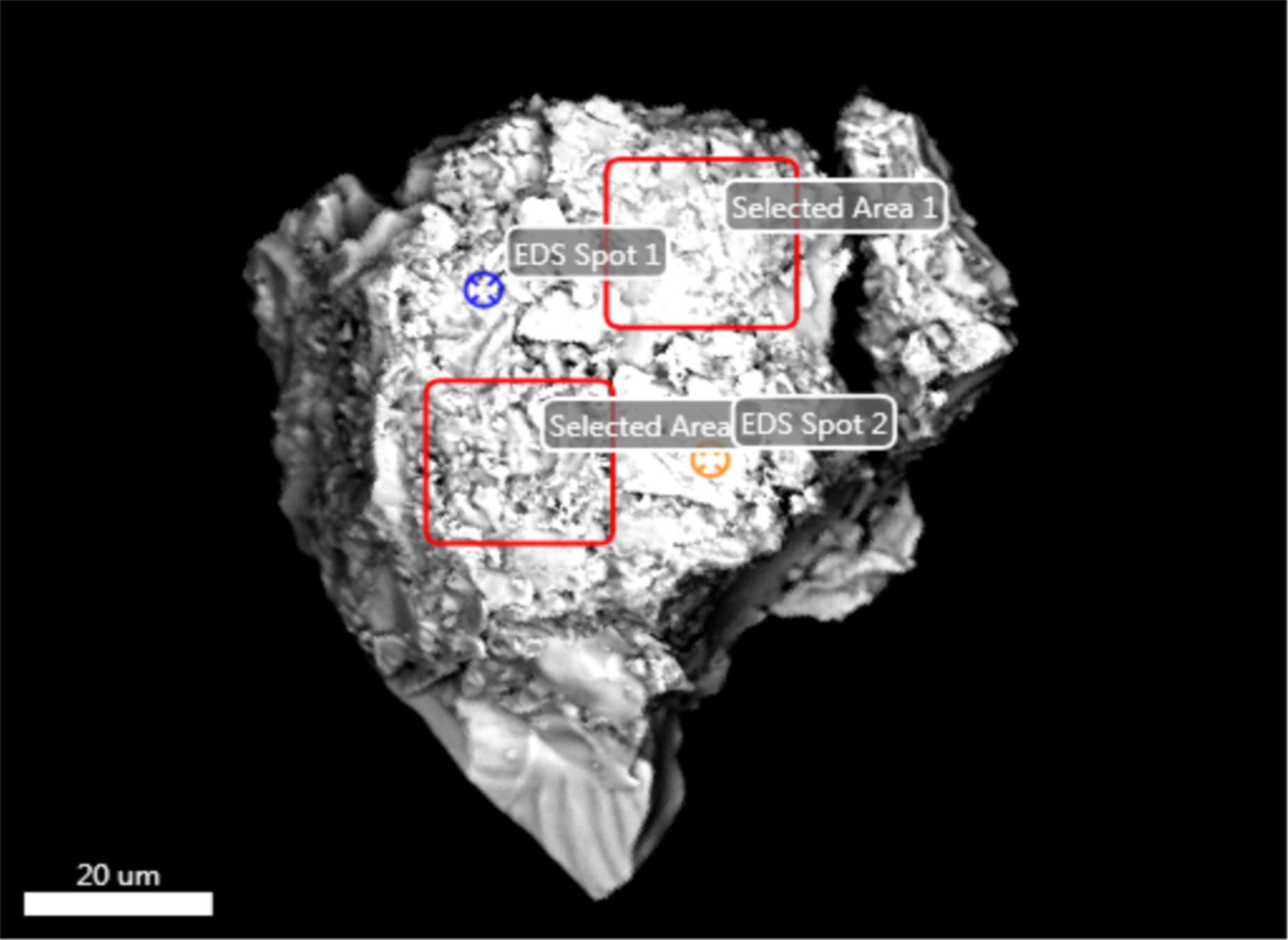


Fig. S2 Sample 2 with selected spots and areas for EDX analysis

**Table S2 EDX results for selected points and areas as designated in Fig. S2**

|  | Element | Weight (%) | Atomic (%) | Error (%) | Ti : Fe : C : O |
| --- | --- | --- | --- | --- | --- |
| Spot1 | TiK | 68.41 | 60.09 | 1.55 | 6.198:2:2.12:× |
|  | FeK  C K | 25.73  5.86 | 19.39  20.52 | 2.81  9.32 |  |
| Spot2 | TiK | 7.45 | 6.28 | 3.16 | 0.214:2:0.90:0.30 |
|  | FeK  C K  O K | 81.26  7.82  3.47 | 58.71  26.28  8.74 | 1.74  10.13  9.36 |  |
| Area1 | TiK | 50.98 | 39.75 | 1.66 | 3.32:2:1.82:1.21 |
|  | FeK | 35.79 | 23.94 | 2.44 |  |
|  | C K  O K | 7.01  6.22 | 21.79  14.52 | 9.21  11.55 |  |
| Area2 | TiK | 55.27 | 41.38 | 1.59 | 4.30:2:2.64:1.45 |
|  | FeK | 29.99 | 19.26 | 2.57 |  |
|  | C K  O K | 8.50  6.23 | 25.39  13.97 | 8.92  11.71 |  |

**Table S3** Different options of refinement and the resulting refined chemical compositions

|  | location | | | | | | | compositions | | | | | | | | | R_1_ | |  |  |
| --- | --- | --- | --- | --- | --- | --- | --- | --- | --- | --- | --- | --- | --- | --- | --- | --- | --- | --- | --- | --- |
| Ti_4_Fe_2_C_0.82_O_0.18_ | 16*c* | 32*e* | | 48*f* | | 16*d* | | | | Ti | Fe | | C | | O | | |  | |  |
|  | **Ti1** | **Fe** | | **Ti2** | | **C/O** | | | | **63.9994** | **32.0006** | | **13.1382** | | **2.86117** | | | **2.18** | |  |
|  | Ti1 | Fe | | Ti2 | | C | | | | 63.9994 | 32.0006 | | 15.9994 | |  | | | 2.24 | |  |
|  | Ti1 | Fe | | Ti2 | | Partial C | | | | Occupancy error | | | | | | | |  | |  |
|  | Ti1 | Fe | | Ti2 | | O | | | | NPD atoms | | | | | | | |  | |  |
|  | Ti1 | Fe | | Ti2 | | Partial O | | | | 63.9994 | 32.0006 | |  | | 11.0396 | | | 2.14 | |  |
| Ti_4_Fe_2_C_0.87_O_0.13_ | 16*c* | | 32*e* | | 48*f* | | 16*d* | | Ti | | | Fe | | C | | O | |  | | |
|  | **Ti1** | | **Fe** | | **Ti2** | | **C/O** | | **63.9994** | | | **32.0006** | | **13.9202** | | **2.07912** | | **2.48** | | |
|  | Ti1 | | Fe | | Ti2 | | C | | NPD atoms | | | | | | | | |  | | |
|  | Ti1 | | Fe | | Ti2 | | Partial C | | Occupancy error | | | | | | | | |  | | |
|  | Ti1 | | Fe | | Ti2 | | O | | 63.9994 | | | 32.0006 | |  | | 15.9994 | | 3.21 | | |
|  | Ti1 | | Fe | | Ti2 | | Partial O | | NPD atoms | | | | | | | | |  | | |

**Table S4** Experimental details of Ti_4_Fe_2_C_0.87_O_0.13_ phase

|  | Ti_4_Fe_2_C_0.87_O_0.13_ |
| --- | --- |
| Crystal data | |
| Chemical formula | Ti_4_Fe_2_C_0.87_O_0.13_ |
| *M*_r_ | 315.83 |
| Crystal system, space group | Cubic, *Fd*$\bar{\text{3}}$*m* |
| Temperature (K) | 296 |
| *a* (Å) | 11.326 (4) |
| *V* (Å^3^) | 1453.1 (15) |
| *Z* | 16 |
| Radiation type | Mo-K*α* |
| µ (mm^-1^) | 15.89 |
| Crystal size (mm) | 0.08×0.08×0.05 |
| Data collection | |
| Diffractometer | Bruker D8 Venture Photon 100 COMS |
| Absorption correction | multi-scan  (SADABS; Krause *et al*., 2015) |
| *T*_min_, *T*_max_ | 0.275, 0.746 |
| No. of measured, independent and  observed [*I* > 2σ(*I*)] reflections | 6013, 84, 74 |
| *R_int_* | 0.161 |
| (sin θ/λ)_max_ (Å^−1^) | 0.592 |
| Refinement | |
| *R*[*F*^2^ > 2σ(*F*^2^)], *wR*(*F*^2^), *S* | 0.025, 0.048, 1.25 |
| No. of reflections | 84 |
| No. of parameters | 13 |
| Δ*ρ*_max_, Δ*ρ*_min_ (e Å^−3^) | 0.53, -0.75 |

Computer programs: *APEX3* (Bruker, 2015), *APEX3* and *SAINT* (Bruker, 2015), SHELXT 2014/5 (Sheldrick, 2015), *SHELXL2016*/6 (Sheldrick, 2015), *publCIF* (Westrip, 2010).
